# What the salamander eye has been telling the vision scientist’s brain

**DOI:** 10.1016/j.semcdb.2020.04.010

**Published:** 2020-10

**Authors:** Fernando Rozenblit, Tim Gollisch

**Affiliations:** a Department of Ophthalmology, University Medical Center Göttingen, 37073, Göttingen, Germany; b Bernstein Center for Computational Neuroscience Göttingen, 37077, Göttingen, Germany

**Keywords:** Retina, Vision, Ambystoma, Salamander, Mudpuppy, Axolotl

## Abstract

Salamanders have been habitual residents of research laboratories for more than a century, and their history in science is tightly interwoven with vision research. Nevertheless, many vision scientists – even those working with salamanders – may be unaware of how much our knowledge about vision, and particularly the retina, has been shaped by studying salamanders. In this review, we take a tour through the salamander history in vision science, highlighting the main contributions of salamanders to our understanding of the vertebrate retina. We further point out specificities of the salamander visual system and discuss the perspectives of this animal system for future vision research.

## Highlights

• Salamanders have a long history as laboratory animals.
• Their retinal cells are large and great targets for characterizations and recordings.
• Salamander studies elucidated many retinal cells, circuits, and functions.
• New approaches may come from emerging genetic toolkits for the axolotl.

## Graphical abstract

**Figure:**
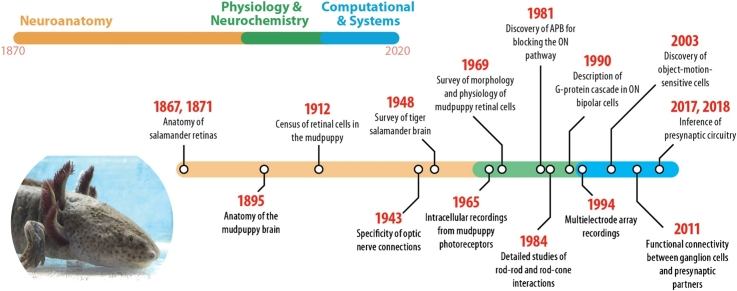

## 1 Introduction

“Why salamander?” If you are a neuroscientist working with the salamander visual system, this may well be the most common question that you hear after presenting your work at an outside talk. And if you are not, you may have asked this question yourself when coming across one of the surprisingly many works in visual neuroscience built on investigating these animals. How indeed have these cold-blooded, egg-laying amphibians, which spend a great deal of their lives in water and are distant from us humans by more than 300 million years of separated evolution [1] come to be a model for studying the early visual system?

It is this question that we focus on in this review. We take a historical tour that highlights key contributions that salamanders have brought to our understanding of the early visual system. These contributions have been successively built upon each other and have often used two key properties of the salamander nervous system: particularly large neurons and extraordinary robustness to experimental manipulations. We find general concepts about the visual system that have emerged from work on the salamander, as well as peculiarities that are of interest for comparative and ethological studies. Finally, we ask what future role the salamander plays for vision research. Throughout this tour, our focus will be on the retina, the neural network at the back of the eyeball where the first stages of visual processing in vertebrates occur. This is where the salamander has had an outstanding influence on the field of vision science. Altogether, the body of work on the salamander visual system is truly immense, and we necessarily had to leave out many important works; there is no pretension of completeness of this overview.

## 2 The order of salamanders

Salamanders, together with newts, form the amphibian order Urodela. The other two amphibian orders are Anura (frogs and toads) and Apoda (the limbless and mostly blind caecilians). All amphibians can be considered evolutionarily early vertebrates. Relatively soon after the first tetrapod vertebrates started treading dry land, amphibians separated from what would become reptiles, birds, and mammals. These latter groups experienced radical changes in body plan [2] that allowed more complex patterns of locomotion and the occupation of new ecological niches. Concomitantly, brain areas enlarged, differentiated and gave rise to new structures such as the cortex. Many amphibians, on the other hand, did not undergo such drastic changes. Urodeles, in particular, seem to have kept close to their original lifestyle and are thus considered to occupy an intermediate step in evolution, with brains lacking a cortex and displaying an anatomy that may resemble those of the first land-dwellers [3].

Salamander brains are relatively simple [3,4] even when compared to those of other amphibians or lampreys and hagfishes, suggesting a certain phylogenetic simplification [5]. For instance, the salamander tectum shows little lamination and only 30,000–90,000 cells, compared to the 800,000 in the tectum of anurans [5]. Nevertheless, salamanders can see – and process what they see – well enough to help them flee, feed, and procreate [6]. Both larvae and adults are carnivorous and need to hunt. Some species, like the tongue-projecting salamanders (genus Bolitoglossa), have been shown to depend on vision for determining the distance to prey quickly and precisely [7]. Others, like the tiger salamander (*Ambystoma tigrinum*), which despite its name prefers to sit and wait for its prey, rely on vision for deciding when to strike [8].

### 2.1 Diversity of species

Salamanders comprise more than 700 species [9] and are overall very diverse. While it is commonly thought that salamanders start their life as larvae in water until metamorphosing into a terrestrial adult form, this view is incorrect for two thirds of salamander species [10]. In the lungless salamander family (Plethodontidae), the most speciose, animals hatch directly from eggs into a terrestrial form [11]. Other species, like *Necturus maculosus* (mudpuppy) and *Ambystoma mexicanum* (axolotl), display neoteny: individuals can reach sexual maturity in their larval forms and may never metamorphose [12].

**Fig. 1:**
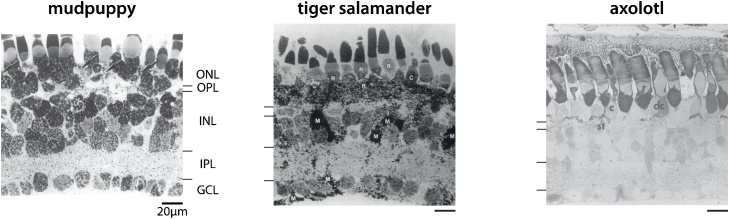
Cross-section of the retina for three salamander species. From top to bottom in each cross-section: outer nuclear layer (ONL), outer plexiform layer (OPL), inner nuclear layer (INL), inner plexiform layer (IPL), and ganglion cell layer (GCL). Thin lines indicate the borders between layers. Mudpuppy, tiger salamander, and axolotl retinas are structurally alike with large cells and thin plexiform layers. Mudpuppy and tiger salamander retina cross-sections are autoradiographic and adapted respectively from [203], Copyright (1984) and [204], Copyright (1997) with permission from Elsevier. Axolotl retina cross-section from light microscopy adapted from [50] with permission (John Wiley and Sons, Copyright 1973 The Wistar Institute of Anatomy and Biology).

With so many species of salamanders, it is no wonder that vision has been studied in many of them. And although findings are often treated as coming from a single type of animal (and we here may do the same for expediency when the context is clear), it is important to note that there really is no “the salamander” as a species in vision research. Yet, three species have contributed dearly to our understanding of vision and thus have a special place in this tour. They are the three darlings of salamander retinal research: *Necturus maculosus* (mudpuppy) and two closely related species of mole salamanders, *Ambystoma tigrinum* (tiger salamander) and *Ambystoma mexicanum* (axolotl). Their retinas display the same, characteristic structure (Fig. 1), with fewer and larger cell bodies as compared to mammalian retinas, which has proved a boon for retina research. Knowing about the characteristics and idiosyncrasies of these species provides an essential context for studying their visual systems.

#### 2.1.1 *Necturus maculosus* (mudpuppy)

Mudpuppies are large, fully aquatic salamanders that have never been observed to metamorphose [12]. Individuals reach over 30 cm in length, become sexually mature at about 5 years of age, and are often found in the region of the Great Lakes in North America [13].

The anatomy of the mudpuppy brain was described in detail more than a hundred years ago by Kingsbury [4]. At the time, the mudpuppy brain was already considered to strike a good balance in size for anatomical investigations at both microscopic and macroscopic levels, a property that was exploited later on for detailed anatomical descriptions of rods and cones [14,15] and for recordings of the retinal output [16,17].

#### 2.1.2 *Ambystoma tigrinum* (tiger salamander)

The genus “Ambystoma” has been plagued with controversy, starting with its name [18]. This genus was first proposed in the early 19th century by Tschudi [19] to refer to North-American mole salamanders. Believing the name to be a misspelling [20,21], some authors took the liberty to rename the genus as “Amblystoma”. The case was only settled after a vote by the International Commission on Zoological Nomenclature in 1963 [22,23].

Tiger salamanders (*Ambystoma tigrinum*) were once considered to be a single species extending over most of North America but are now best divided into several subspecies, each with a specific geographic range [24]. All subspecies of *Ambystoma tigrinum*, as well as multiple closely related species from Mexico (including the axolotl, see below), form the tiger salamander species complex [24]. Some subspecies are facultative paedomorphs while others must metamorphose to reach maturity [25]. Tiger salamanders are the largest mole salamanders, and adults in the wild can be more than 20 cm long [13]. Their brains have been studied in detail as early as the 1940s [3], and over recent years, tiger salamanders have received much attention in vision research and thus have become something like the standard salamander system in the field.

#### 2.1.3 *Ambystoma mexicanum* (axolotl)

**Fig. 2:**
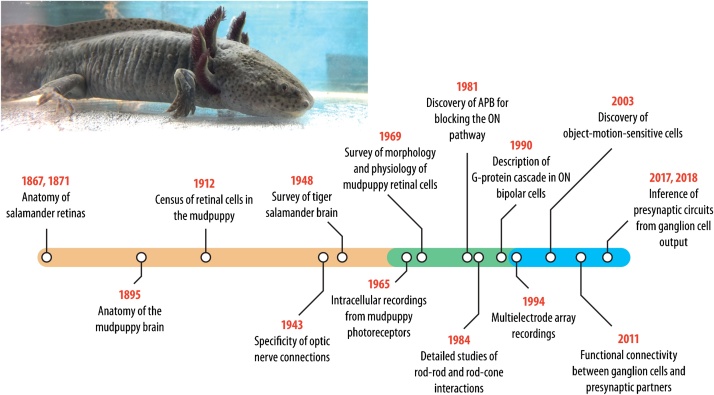
Samples from the salamander tour in vision science. The timeline shows selected contributions of the salamander to vision research and can be roughly divided into a period centered on neuroanatomy (orange region), a period with focus on cellular neuroscience and neurochemistry (green), and a period with major contributions to systems and computational neuroscience (blue). The image on top shows an axolotl (photo kindly provided by Norma Kühn). The fuzzy-looking appendages at the neck are external gills, a typical feature of aquatic salamanders.

Historically, the name axolotl referred to the larval stage of ambystomatid salamanders regardless of species [26]. Nowadays, it is reserved for a single species, *Ambystoma mexicanum*, originally coming from an area near Lake Xochimilco in Mexico [23,27]. In the wild, axolotls are facultative paedomorphs and are known to metamorphose if needed [23]. In laboratories, likely because of artificial selection, axolotls remain in their larval forms (Fig. 2, top) unless hormonally induced to metamorphose [12,23]. The larvae can look strikingly like those of tiger salamanders.

Axolotls have a long history as a laboratory animal [28]. In Europe, the first colonies started with the arrival of 34 live axolotls from Mexico in 1864. Six of these animals were donated to the Paris Natural History Museum, where they reproduced so successfully that their offspring soon dispersed to other European institutions. Most laboratory axolotls nowadays are related to these first six axolotls [27].

At the beginning of the 20th century in the United States, Humphrey started a colony with many well-characterized axolotl mutants [29]. This included strikingly white axolotls, with reduced skin pigmentation but pigmented eyes. However, the absence of a true axolotl albino and the discovery of a tiger salamander albino in the wild led Humphrey to create a hybrid of a white axolotl with this albino tiger salamander [30]. The hybrid offspring were crossed into various axolotl strains, kept by the Ambystoma Genetic Stock Center (AGSC) at the University of Kentucky, an important supplier of axolotls for research, and left their lasting genetic mark, perhaps by being particularly fertile. Indeed, most axolotls in the AGSC are now an ambystomatid hybrid containing about 6% of tiger salamander DNA [31].

An interesting mutant is the eyeless axolotl. First observed as a spontaneous occurrence in a stock [32], these mutants lack eyes due to a developmental defect [33]. Yet, transplanting eyes from a regular axolotl to an eyeless one at an early developmental stage can recover visual object localization and the optokinetic reflex as well as normal vision-driven skin pigmentation [34,35]. Indeed, nerve fibers from the transplanted eye manage to find their usual target areas, though through unusual paths that can differ from animal to animal [33,35].

### 2.2 Convenience as a model system in early vision research

There are probably two aspects that explain why salamanders early on became such a well-studied system in vision research. On the one hand, their nervous system appears to be particularly robust to handling and manipulations [[36], [37], [38], [39]], allowing experiments and functional studies that might be more difficult in other animal systems.

**Fig. 3:**
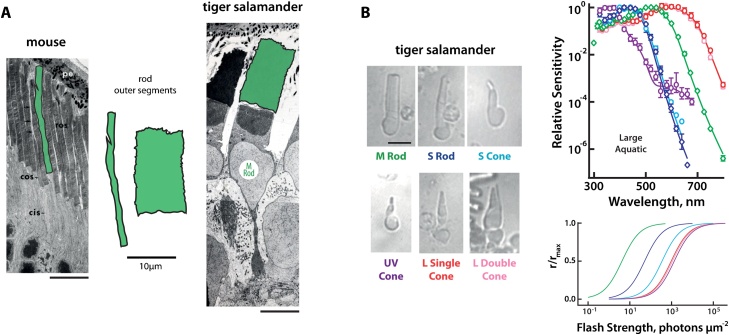
Tiger salamander photoreceptors. (A) Size comparison of rod photoreceptors in mouse and tiger salamander. Outer segment regions are highlighted with green shading and reproduced in the center for direct comparison. Scale bars: 10 μm. Original electron microscope images adapted from [41] (mouse) with permission (John Wiley and Sons, Copyright 1979 The Wistar Institute Press) and from [40] (tiger salamander) with permission (Copyright 1986 The Royal Society). (B) Six types of tiger salamander photoreceptors. The figure shows morphologies (left) as well as sensitivity to wavelength (right, top) and to flash intensity (right, bottom; obtained at preferred wavelength and normalized to peak response). Adapted from [121] with permission (John Wiley and Sons, Copyright 2013 Wiley Periodicals Inc.).

On the other hand, and perhaps most importantly, salamanders turn out to have notably large cells. Their photoreceptors, for example, can have outer segment diameters of 10−13 μm [40], considerably larger than the 1−2 μm of typical mouse photoreceptors [41] (Fig. 3A). But large cells can be found throughout the salamander’s nervous system and indeed throughout their entire body [42]. This seems to be at least partly a consequence of their large genome, requiring a large nucleus to accommodate it [43,44]. The axolotl, for example, carries 34.75 picograms (pg) of DNA per haploid genome [44], whereas typical vertebrate haploid genomes contain less than 7 pg DNA [43]. The mudpuppy genome even amounts to a staggering 83 pg DNA per haploid genome. Interestingly, genome size among salamander species is negatively correlated with brain complexity, indicating that larger cells may imply simpler brains [44]. A curious side-effect of large cells for vision research is that the focus of visual stimuli on the large photoreceptors does not need to be so precise. For the animal, this means that less accommodation is required from the lenses, allowing for simpler eyes [45]; for the vision researcher, this means easier control of visual stimuli.

## 3 A long history of contributions to retina research

### 3.1 Structural studies

There is a long history of using salamanders for investigating the retina (Fig. 2), which considerably helped advance our basic understanding of the retina’s structure and function. Even some of the very first studies of retinal organization were already performed with salamanders [46,47]. The large cells of the mudpuppy allowed general descriptions of rods and cones [14] as well as a count of all cells in a single retina [48], leading later to one of the first and most detailed structural characterizations of photoreceptors [15]. The salamander retina also contributed to revealing electrical gap junctions in the retina, which had been proposed to explain signal spread between neighboring cones in electrophysiological experiments [49]. Observing junctions in electron microscopic examinations of the axolotl [50] and tiger salamander [51] retina then provided structural evidence for electrical connections between photoreceptors as well as between horizontal cells.

### 3.2 Synapses and signal transmission

Salamanders were also present as the first electrophysiological investigations of the retina were performed. Already Hartline – in his seminal studies of single optic nerve fibers, which led to his eventual Nobel prize – recorded from the mudpuppy, though his amphibian work mostly focused on frogs [52]. For the next few decades, the mudpuppy retina – thanks to the large cells that allowed intracellular recordings [53] – was one of the most widely studied early vision systems, used to show the match of morphology and physiology for the different retinal cell classes [17], to characterize light and dark adaptation [54,55], to reveal the different kinetics of rods and cones to flashes of light [56], and to elucidate the role of amacrine cells in lateral inhibition [57].

The mudpuppy retina also played an essential role in dissecting the ON and OFF pathways in the retina. The possibility to record intracellularly from all retinal cell types in the mudpuppy retina in chloride-free solutions [58], where ON responses are suppressed, revealed how signals flow from ON and OFF bipolar cells to ON, OFF, and ON-OFF ganglion cells [59]. Shortly after, intracellular recordings in the mudpuppy retina were used to demonstrate that 2-amino-4-phophonobutyric acid (APB or also AP4) selectively blocks ON bipolar cells [60], suggesting unique receptors in ON bipolar cells as well as providing a pharmacological tool that is still widely used today in retina research. Later recordings in the tiger salamander, combined with pharmacological interventions, revealed the G-protein-mediated cascade in ON bipolar cells that leads to the closure of a cation channel upon receptor activation [61].

Combining intracellular recordings in the mudpuppy retina and pharmacological blockade of synaptic signals furthermore provided essential steps in elucidating that glutamate is the neurotransmitter released by photoreceptors [62] as well as by ON and OFF bipolar cells [63] and in revealing the role of NMDA receptors in channeling signals through the retina [64]. And, patch-clamp recordings from tiger salamander rods and their postsynaptic partners demonstrated that the bandpass filtering in this synaptic signal transmission supports the detection of dim light near absolute darkness [65].

### 3.3 Circuits, computations, and coding

With so much fundamental insight about retinal organization and synaptic mechanisms coming from the salamander retina, it is no wonder that the system was also used early for functional investigations that asked how the retinal network processes and encodes visual information. Besides the accessibility for intracellular recordings, these investigations benefitted from outstanding robustness and longevity of isolated retinal tissue with intact light responses. This proved advantageous for recording ganglion cell spiking activity with the emerging multielectrode arrays [66]. The possibility to monitor the activity of many ganglion cells simultaneously over a long time under visual stimulation allowed detailed explorations of the retina’s neural code and computations, with principles often first identified in the salamander and later confirmed in other systems. The salamander retina thereby became an essential tool for developing and testing new approaches that helped shape the field of systems and computational neuroscience.

Multielectrode-array recordings, mostly from tiger salamander retina, revealed how the retina adapts to visual contrast [[67], [68], [69]] as well as to more complex spatiotemporal stimulus structures [70,71] and chromatic components [72], with intracellular recordings adding mechanistic insights [[73], [74], [75]]. Such recordings also revealed contrast sensitization in the retina, that is, the increase rather than decrease of sensitivity in some cells under increased visual contrast [76]. Another thought-provoking adaptation discovered through the salamander retina is the “omitted stimulus response”, characterized by the entrainment to a periodic sequence of stimulus pulses and the occurrence of activity bursts when the sequence has ended or when a pulse has been omitted [77,78].

There are also important contributions that helped revise our view of retinal receptive fields, for example, by showing that ganglion cells can be transiently turned through peripheral stimulation from being OFF cells to preferring ON-type contrast [79], that different types of nonlinearities can shape how stimuli are integrated in the receptive field center [80] and surround [81], and that cells can encode motion stimuli far outside their receptive field center [82]. These and other findings in the salamander retina helped shape the emerging view that specific retinal circuits can execute specific visual functions [[83], [84], [85]], such as the distinction between global and differential motion by object-motion-sensitive cells [86,87] or the cancelation of processing delays for predicting the location of a moving object [88,89].

The possibility to record activity from many ganglion cells simultaneously for long durations with multielectrode arrays also spurred the analysis of retinal population codes, for example, by suggesting that synchronized multineuronal spiking may provide a rich, combinatorial neural code [90,91]. Synchronization of ganglion cells was furthermore shown to occur in the salamander under rapid periodic stimulation that induces period doubling, which provided a model for similar observations in human electroretinograms [92]. Later, statistical analyses of synchronization among salamander retinal ganglion cells revealed strongly ordered collective activity in large cell populations [93], which may facilitate stimulus discrimination [94,95]. Ganglion cell synchronization may also be of particular importance for motion encoding as shown by recordings from tiger salamander and axolotl, providing an error signal when an object suddenly reverses direction rather than continuing straight on its path [96,97] and allowing to disentangle motion-direction-related from contrast-related activity in populations of direction-selective ganglion cells [98]. Furthermore, spike timing differences in near-synchronous salamander ganglion cell activity has been shown to provide a rapid code for suddenly appearing visual images [99].

Methodologically, a particularly interesting extension of the multielectrode-array recordings is the possibility to combine them with simultaneous intracellular recordings from cells presynaptic to the ganglion cells. This, again, is aided by the relatively large bipolar and amacrine cells of the salamander retina and has allowed direct investigations of the connectivity between these interneurons and their ganglion cell targets [[100], [101], [102]]. A more recent avenue is to use new computational resources and tools to perform such circuit analysis through computationally demanding inference methods or model fitting, for example, to reveal the layout and dynamics of presynaptic bipolar cells from ganglion cell recordings [[103], [104], [105]]. This also continues the use of the well-controlled and reliable data that can be obtained in recordings from salamander retina as a testbed for novel techniques in computational data analysis. Earlier examples for this are stimulus reconstruction from multi-neuronal activity [106], spike-feedback models to capture the precision and reliability of spiking events [107], and applications of multi-filter models for stimulus-response relations [108,109]. It therefore comes as no surprise that this system is among the first where the new ideas of using deep learning in neural networks have been used successfully to model neuronal signal processing [110].

## 4 Salamander retina specifics

The previous section has highlighted the use of the salamander retina as a beneficial system for studying general features of the retina. Yet, interesting insights also come from differences to other animals, and investigating the salamander retina has certainly provided a rich set of specifics and idiosyncrasies that distinguish it from mammals or other vertebrates. Some of these we discuss in this section.

### 4.1 Detection of light

Rods and cones are the light sensing cells of the retina. Most vertebrates share a similar set of photopigments since those first appeared around 500 million years ago [111]. Regarding salamanders, photoreceptors have been most thoroughly described in tiger salamanders, which have six types (Fig. 3B), comprising two rods and four cones [40,112]. In total, rods and cones are almost equally numbered in the larval tiger salamander retina, with cones slightly outnumbering rods near the center and vice versa in the periphery [113]. Among the rods, the vast majority is tuned to medium wavelengths (M-rod), with highest sensitivity for green light. The other rod type only comprises a few percent of the rods and is smaller and tuned to short wavelengths (S-rod). The presence of two rods is common in amphibians [114]. Because the rods were first distinguished (in frogs) based on their apparent color under a microscope, the M- and S-rods are also (perhaps confusingly) referred to as “red” (green-absorbing) and “green” (blue-absorbing) rods [115].

Most cones (85 %) in the salamander retina, the single and double L-cones, express a long-wavelength opsin [112]. Double L-cones are composed of two tightly attached cones, a principal and an accessory cone. The remaining UV- and S-cones are almost equal in number. No medium-wavelength-preferring cones were identified [40,112]. Axolotls are thought to have similar photoreceptor distributions, including UV cones [116]. Mudpuppies, on the other hand, exhibit a simpler layout with potentially only one rod and two cone types [114,117]. Despite the rich set of photoreceptor types in some salamanders, little is known about whether these animals have color vision, except that one species (*Salamandra salamandra*) appears to use differences in color to guide behavior [118,119].

Interestingly, S-cones and S-rods in the tiger salamander share the same opsin, but S-rods have more pigment, which may explain their higher sensitivity to flashes [120] (see Fig. 3B). Furthermore, UV- and single L-cones, as well as the accessory member of the double cones, express more than one opsin. Besides their primary opsins that determine their peak sensitivity, UV-cones express low amounts of S- and L-opsins, while the single L-cones and the accessory member of the double L-cones express UV- and S-opsins. The exact pigment ratios in L-cones may differ from cell to cell, but UV- and S-pigments can comprise up to a third of all pigments in some L-cones [[121], [122], [123]]. The ethological relevance of this opsin co-expression is yet to be shown. Perhaps it helps when achromatic detection of light is desirable; for instance, when detecting prey against a brightly lit background [121].

Rods are coupled to neighboring photoreceptors. In the axolotl and tiger salamander, there is evidence for gap junctions from rods to other rods and cones [40,50] but no direct connections have yet been found between cones. Each rod is typically coupled electrically to four other rods and four cones [124]. Some rods are so strongly coupled to cones that they change their spectral sensitivity with changes in background illumination [125].

### 4.2 Signal transmission from photoreceptors to bipolar cells

Bipolar cells in the salamander retina have dendritic trees with diameters ranging from 50 to over 100 μm [126], considerably larger than, for example, in mouse retina. Surveys of bipolar cells in tiger salamander retina [[126], [127], [128]] distinguished at least 12 different types, based on functional and morphological properties. In general, salamander OFF bipolar cells are observed to be about 30 ms faster in their response kinetics than ON bipolar cells [129]. Curiously, it has been reported that one bipolar cell type, which stratifies in two layers of the inner plexiform layer, may possess both ON-type and OFF-type response properties, perhaps depending on light levels [127].

It has been realized early on for the salamander retina that rods as well as cones make direct synapses to multiple types of bipolar cells [51]. For both ON and OFF bipolar cells, rod-dominated as well as cone-dominated types can be found [130], with rod-dominated bipolar cells stratifying preferentially at the two edges of the inner plexiform layer and cone-dominated bipolar cells more centrally. It is worth noting that the interconnectedness of rod and cone signals at the level of bipolar cells originally appeared to be a striking difference from the mammalian retina, which contains distinct rod and cone bipolar cells. Meanwhile, however, evidence has been accumulating that, at least in mouse, the rod bipolar cell and some cone bipolar cells also receive input from those photoreceptors that are not part of their name [[131], [132], [133]], making this distinction between salamander and mammalian retina more gradual than absolute.

Morphologically, an interesting feature of salamander bipolar cells is the occurrence of a Landolt club [47], a protrusion of the cell, potentially rich in mitochondria and extending towards the photoreceptor cell bodies similar to dendrites but without synaptic contacts [134]. Landolt clubs are observed in most, if not all, bipolar cells in amphibians [51], as well as in some other non-mammalian species.

### 4.3 Inhibitory interactions

The information flow through the retina from photoreceptors via bipolar cells to ganglion cells is modulated by inhibitory signals from horizontal and amacrine cell [135]. Horizontal cells come in two types in the tiger salamander [136,137]. One of the two types has two distinct regions of neurite branching, coupled by a thin axon, providing in total three potentially distinct horizontal cell processing entities, with differences in relative rod versus cone inputs, receptive field sizes, and gap junction coupling [137].

Amacrine cells in the tiger salamander release the conventional inhibitory neurotransmitters GABA and glycine as well as the neuromodulators dopamine and serotonin [[138], [139], [140], [141]]. In addition, some amacrine cells appear to be cholinergic [142,143], which, in the mammalian retina, is usually associated with the circuit of direction-selective ganglion cells, though a similar function of cholinergic salamander cells has not yet been shown.

Somewhat of a controversy exists about whether amacrine cells in the salamander follow the same relation of neurotransmitter to size as observed in the mammalian retina, where GABAergic amacrine cells are mostly large, wide-field or medium-field cells and glycinergic ones mostly narrow-field [144]. Studies in retinal slices of the tiger salamander indicated longer interaction distances for glycinergic as compared to GABAergic amacrine cells [145] and mostly wide-field characteristics of glycinergic cells [146], suggesting that the neurotransmitter-to-size relation may be opposite to that in mammals [147]. However, later analyses of amacrine cells in whole-mount preparations found mostly wide-field GABAergic cells and narrow-field glycinergic cells [142], in accordance with the mammalian system. Thus far, this question remains unresolved.

Bipolar cells mostly express GABA_C_ receptors at their synaptic terminals [148]. Here, the release of glutamate can be modulated by GABAergic amacrine cells [149], which may enhance the temporal contrast at the terminals [150]. There also have been observations of glycine receptors at the dendrites of bipolar cells [140], though they don’t appear to contribute to the receptive field surround [151]. These glycine receptors may be the target of glycinergic interplexiform cells, which have been shown to affect the dendrites of bipolar cells [152], perhaps to regulate the gain of signal transmission between photoreceptors and bipolar cells [153].

Interplexiform cells form a class of retinal neurons that receive input at the inner retina, resembling amacrine cells, but stratify at the outer plexiform layer and are thought to provide feedback across the synaptic layers [154]. At least three morphological types of interplexiform cells have been described in the tiger salamander [155]. They are all spiking cells, receive ON as well as OFF sustained excitation from bipolar cells, and release GABA or glycine [140]. Dopaminergic interplexiform cells, which have been found in other animals such as frog [156], appear to be absent or extremely rare in the salamander retina [139].

### 4.4 Displaced amacrine, bipolar, and ganglion cells

Retinal neurons are typically located according to their type in one of the three retinal nuclear layers. There are, however, exceptions to this ordered organization. Displaced amacrine cells, for example, are found in the ganglion cell layer rather than in the inner nuclear layer where most amacrine cells are situated. In the tiger salamander ganglion cell layer, about one quarter of cells are displaced amacrine cells [157]. In addition, there are displaced bipolar cells in the layer of photoreceptor cell bodies (outer nuclear layer) and displaced ganglion cells among the layer of amacrine and bipolar cells (inner nuclear layer).

Displaced bipolar cells are mostly OFF cells and account for about 17 % of the somas in the outer nuclear layer among the photoreceptors [126,128]. Estimates suggest that almost 45 % of the OFF bipolar cells [128] and 5–15% of the ganglion cells may be displaced in the salamander retina [[158], [159], [160]]. Note, though, that displaced ganglion cells have also been reported in many other species, including mice [161] and monkeys [162].

### 4.5 Output channels

The visual information extracted by the retina is encoded into the spiking activity of ganglion cells, the retina’s output channels. Unlike in the mammalian retina, the population of ganglion cells in the salamander retina is dominated by OFF cells and ON-OFF cells [[163], [164], [165]], a property that is shared, for example, by frog and turtle retina. In tiger salamander, true ON-type ganglion cells may be as few as 5%, and ON-OFF cells may make up around two thirds of ganglion cells, often with a bias towards OFF-type responses [163]. While the majority of these ON-OFF cells receive excitatory input from both ON and OFF bipolar cells, some ganglion cells seem to obtain their ON-OFF responses from release of inhibition mediated by amacrine cells with hyperpolarizing responses to both light onset and offset [166].

Ganglion cells come in many types, which further divide the broad classes of ON, OFF, and ON-OFF cells. Distinguishing these types based on neuron morphology, response characteristics to visual stimuli, or both is an ongoing research direction, and no consensus exists yet on the number of different types or their characteristic features. Several reports, including early morphological studies [160], functional investigations of light responses [164,165], and combinations of morphology and function [157], identify at least five to seven types in the tiger salamander. Important criteria in these classifications are the size and symmetry of the dendritic tree, the dendritic stratification in the inner plexiform layer, the relative contributions of rod- and cone-driven inputs, and the filtering kinetics for visual stimuli.

Surprisingly, however, there is still little information about to what extent the identified ganglion cell types in the salamander tile the visual space with their receptive fields. This tiling is considered a tell-tale sign of having identified a distinct type of ganglion cells; like shards in a stained-glass mosaic, receptive fields of single ganglion cell types are expected to cover the visual field with little overlap. Clustering analyses of functional ganglion cell types in the salamander, however, identified only one type with tiling receptive fields, whereas other types showed considerable overlap [164,165], leading to the speculation that tiling may not be a general property of ganglion cells in the salamander. Later, however, further examples of tiling for specific types of salamander ganglion cells have surfaced (though not in the context of general classification studies), when additional response characteristics were considered, such as adaptation [76] or direction selectivity [167]. It thus remains to be seen whether enhanced classification methods might provide a refined separation of recorded ganglion cells into perhaps a larger number of types with tiling receptive fields.

A functional class of ganglion cells of widespread interest is the class of direction-selective cells. These cells respond to a specific direction of visual motion, but are suppressed by the opposite direction [168]. Yet, for the salamander, investigations of direction selectivity were conspicuously absent, despite early examples in the mudpuppy [[169], [170], [171]] and tiger salamander retina [172], until resurfacing in recordings from axolotls [167]. Unless specific subspecies of salamanders indeed do not possess direction-selective ganglion cells, one may speculate that the lack of reported direction selectivity in surveys of ganglion cell types indicates the need to explore wider ranges of stimulus size and speed or that direction-selective cells in some salamanders are not picked up by multielectrode-array recordings, perhaps because they are not located at the retinal surface and might even be among the displaced ganglion cells.

## 5 Beyond the retina

The visual information encoded by retinal ganglion cells reaches different areas of the salamander brain via the ganglion cell axons, which form the optic nerve. The primary target areas are the optic tectum, the thalamus, the pretectum, the basal optic nucleus, and the hypothalamus [45]. The anatomical layout of the optic tracts that connect the retina to these areas and of the brain regions involved in visual processing are described in detail elsewhere [6,45,173].

Regarding visual signal processing, much less is known about these brain regions as compared to the retina. Most investigations have focused on the optic tectum, and we here only provide few examples. Early recordings in the fire salamander (*Salamandra salamandra*) found that many tectal neurons respond particularly well to moving stimuli, with some showing direction selectivity [174]. It then became a question of particular interest whether these neurons display a stimulus preference that matches the salamander’s prey capture response, which is preferentially triggered by horizontally elongated shapes moving along the horizontal direction, at least at low velocities [175]. Recordings in different salamander species, however, found a variety of shape tunings in individual neurons that generally did not match the behavioral preference [176,177], suggesting a more complex representation of prey stimuli in the tectum [178]. Later recordings in the red-legged salamander (*Plethodon shermani*) with prey-like stimuli indicated that processing in tectal neurons involves feedback from other brain areas and integration of visual information over ranges much larger than classical receptive fields [179].

The ability to test visual behavior through prey-like stimuli also helped establish the importance of ordered connectivity of nerve fibers with their downstream targets. At first, observations that salamanders (and other amphibians) could recover vision after eyes had been excised and grafted back into the eye socket [36,37] had been taken as evidence that neural plasticity in central areas upon regeneration of the optic nerve was so potent as to make specific connectivity unnecessary. However, Roger Sperry – another eventual neuroscience Nobel laureate who appreciated the robustness and simplicity of salamanders – then showed that rotating the eyes of newts either while keeping the optic nerve intact [38] or during grafting after enucleation [39] led to inverted vision. Animals turned away from prey stimuli and displayed an inverted optokinetic reflex. These effects remained over several months, indicating a lack of plasticity. Thus, Sperry concluded that orderly, retinotopic connectivity is essential and that this may be (re-)established by (chemical) signals that are carried by the nerve fibers, which became known as the chemoaffinity hypothesis [180].

The stereotypic, reflex-like visuomotor responses [181] of salamanders have inspired models that capture the animals’ movement and behavior [[182], [183], [184]]. For instance, sensorimotor models of saccades [7,182] can explain intricate behaviors of tongue-projecting salamanders while pursuing prey, like the tendency to meander when one of their eyes is covered [6,182]. Despite the apparent simplicity of visually guided behavior in the salamander, recent investigations have shown surprisingly complex aspects. Tiger salamanders, for example, can learn to use visual cues to solve a T-maze task [185]. And tongue-projecting salamanders can distinguish quantities of prey objects [186] and extrapolate continuous motion to compensate for sensory processing delays [187].

## 6 Open questions and modern developments

### 6.1 Comparisons across species and lifestyle

There is an abundance of salamander species living in diverse ecological niches, some with significant terrestrial life. These species had millions of years to specialize their visual system for these niches [7], perhaps developing differences in their retinas. For example, already in 1897, Slonaker mentioned two salamander species (*Salamandra atra*, *Triturus cristatus*) that presented a higher density of visual cells in central areas of their retinas, suggestive of an area centralis [46,188]. Surveys of other species found no area centralis [6,189], and further reports of such specialized regions appear to be lacking in the literature. However, evidence has surfaced of a weak spatial inhomogeneity in the tiger salamander retina, e.g., in the density of photoreceptors and certain amacrine cells [113]. Comparisons across species of such aspects may help us understand how visual systems are adapted to particular environments.

A drastic change in salamander lifestyle comes with the metamorphosis of the aquatic larvae to terrestrial adults. How the visual system adjusts to its new environment is a fascinating question, about which surprisingly little is known. In the retina, the morphology of the inner plexiform layer and the sensitivity of bipolar cells are apparently unaffected [190,191]. On the other hand, S-cones in the tiger salamander degenerate and are replaced by additional S-rods after metamorphosis [192] – possibly as an adaptation to darker environments on land. This exemplifies that the switch from aquatic to terrestrial life provides an intriguing opportunity to study how the visual system adapts to its environmental challenges.

### 6.2 Salamander lines and genetics

The lack of standard lines in amphibians has been a longstanding issue, with most specimens captured in the wild [193,194]. Even for axolotls, despite their tradition as laboratory animals [28] and well-described genetic background of inbred strains [29,195,196], there are no clear, standardized lines available, which could affect reproducibility of scientific findings across laboratories. Thus, it is custom that researchers report the supplier of their animals.

Over the past decade, mice have developed into arguably the primary model system for vision research, owing to the rich genetic toolkit now available for them. Yet, other animal systems may be catching up, and among salamanders, axolotls appear to be in the best position to compete. While slow reproduction had been an issue in the past, optimized protocols have ensured that transgenic axolotls can be more easily obtained [197]. Recently, the complete axolotl genome was assembled [198]. And the interest in limb regeneration [199] has spurred the development of genetic tools [200,201], which could find powerful applications in vision research.

### 6.3 Future of salamanders in vision research

Due to their large cell size, salamanders were extremely convenient at the infancy of retinal research. This benefit may not be as significant nowadays. Nevertheless, the sheer knowledge accumulated about the physiology and morphology of the salamander retina now provides an expedient background for further explorations of the system. Given the ease of use, the opportunity of comparisons across species as well as across metamorphosis, and the anticipated possibility of transgenic salamanders, we expect salamanders to have, after their long and fruitful past, also a prosperous future in vision research.

The future investigations should also contribute to a more general understanding of early visual processing across species [202]. Their comparatively simple nervous system and the link to stereotypic visual behaviors make salamanders a particularly appealing system for comparison with the current standard model systems of mice and primates in order to study which features of early visual processing generalize across vertebrate species and what the scope of species-specific specializations may be. Thus, a better understanding of visual processing in salamanders will likely be conducive to a more general theory of vision than one that is based on only few select model species.

As a system for studying the early visual system, the salamander has had a fascinating tour over the last hundred years. It started with the discovery that the large cells of the salamander’s neural system provide excellent access for experimental investigations. And the rest – as they say – is history. A history that has greatly influenced the fields of neuroanatomy, neurochemistry, neurophysiology, as well as computational neuroscience and should continue leaving its mark.
